# Tear Film Instability and Meibomian Gland Dysfunction Correlate with the Pterygium Size and Thickness Pre- and Postexcision in Patients with Pterygium

**DOI:** 10.1155/2019/5935239

**Published:** 2019-12-03

**Authors:** Ning Li, Tao Wang, Ruixue Wang, Xuanchu Duan

**Affiliations:** ^1^Department of Ophthalmology, The First Affiliated Hospital of Anhui Medical University, Hefei 230022, China; ^2^Aier School of Ophthalmology, Central South University, Changsha 410000, China; ^3^Changsha Aier Eye Hospital, Aier Eye Hospital Group, Changsha 410000, China; ^4^Medical School of Ophthalmology and Otorhinolaryngology, Hubei University of Science and Technology, Xianning 437100, China

## Abstract

**Purpose:**

This study aimed to evaluate the effects of excision on dry eye and meibomian gland dysfunction (MGD) in individuals with pterygium, before and after surgery. It also aimed to investigate how these effects correlate with the size and thickness of the pterygium.

**Subjects and Methods:**

63 eyes from 63 patients with primary nasal pterygium and 45 eyes from 45 healthy volunteers without ocular pathologies were enrolled in this study. 63 eyes from 63 patients underwent pterygium surgery. ImageJ software was used to calculate the pterygium size based on images of the anterior segments. Anterior segment spectral domain optical coherence tomography (SD-OCT) was performed preoperatively to measure the thickness of the pterygium 1 mm anterior to the nasal scleral spur. The ocular surface disease index (OSDI), Schirmer I Test (SIT), and MGD grade were used to evaluate the eyes, and the eyes were imaged using the noninvasive keratograph average tear film breakup time (NIBUTav), tear meniscus height (TMH), meiboscore, and lipid layer grading tools of the Oculus® Keratograph 5M, preoperatively and at 1, 3, and 6 months postoperatively.

**Results:**

The OSDI, NIBUTav, lid margin abnormality, meiboscore, and lipid layer grading values differed significantly in the pterygium patients in comparison with the controls (*p* < 0.01 for all scores). However, the SIT and TMH values were unchanged between the two groups (all *p* > 0.05). Multivariate regression analysis demonstrated that the NIBUTav, meiboscore, and lipid layer grading score was significantly correlated with the pterygium parameters, such as size and thickness. The postoperative OSDI, NIBUTav, lid margin abnormality, and lipid layer grading values improved significantly (*p* < 0.05 for all scores). The SIT, TMH, and meiboscore results did not differ significantly between the pre- and postoperative values (*p* > 0.05). Among the conventional and automated indexes, at 1 month postoperatively, SIT and TMH were significantly correlated with the pterygium parameters, but no correlation was observed at 3 and 6 months postoperatively. The OSDI, NIBUTav, meiboscore, and lipid layer grading values at 1, 3, and 6 months postoperatively were significantly correlated with the pterygium parameters.

**Conclusion:**

Abnormal tear film and meibomian gland (MG) function improved following pterygium excision in the patients with primary pterygium, which was associated with uncomfortable ocular symptoms. Pterygium parameters, such as size and thickness, correlated with the dry eye and MGD indexes in patients pre- and postoperatively, potentially offering a novel strategy for clinical implementation of pterygium excision surgery.

## 1. Introduction

Pterygium is a common ocular surface disease, defined as fibrovascular overgrowth of the Tenon's capsule and bulbar conjunctiva onto the cornea. The incidence of pterygium ranges from 0.7% to 31% [1]. The exact pathogenesis of this injury is complex, and it is not fully understood. Age, hereditary factors, sunlight, chronic inflammation, microtrauma, and heat are possible contributing factors [2]. Due to the lack of effective therapeutic drugs, pterygium excision, combined with autologous conjunctival grafting, has been suggested as the best treatment for this disorder [3].

While there is extensive research regarding how pterygium excision affects refraction and the ocular surface epithelium, there is a lack of information about the correlations between the pterygium parameters and the prognosis of pterygium excision. Some researchers have reported that pterygium could directly result in localized elevation of the conjunctiva and uneven tear distribution, thereby leading to abnormal dry eye and tear dynamics [4]. Pterygium has also been linked to trefoil and other wavefront aberrations, although surgery can effectively correct these issues [5]. It sooner rather than later pterygium excision can reduce the odds of developing residual aberrations. However, Zhang et al. [6] suggested that when pterygium invades the cornea by more than 2.25 mm, surgery is indicated. Therefore, it remains uncertain whether pterygium parameters (e.g., size and thickness) are directly linked to the need for pterygium excision. If these two parameters are linked, the optimal timing for surgical excision of primary pterygium remains unclear.

The symptoms of pterygium are similar to those of dry eye and meibomian gland dysfunction (MGD), including dryness and irritation. Wu et al. [7] detected a significant association between pterygium size and the meiboscore. Similarly, the pterygium transparency index is positively correlated with the meiboscore, but inversely correlated with the average noninvasive tear film breakup time (NIBUTav). Moreover, clinical work has shown that hypertrophic pterygium can be associated with direct palpebral conjunctival contact, leading to compression under the meibomian glands (MGs) [8]. Consequently, this study aimed to investigate changes in tear formation and MG function because these issues have not previously been studied in this context. Therefore, it sought to assess how the pterygium parameters of size thickness are linked to pre- and postoperative ocular discomfort.

## 2. Subjects and Methods

The principles of the World Medical Association of Helsinki were observed for all aspects of this study. The participants were informed of the study's purpose and the potential risks of participating, and they provided informed consent before participating.

This study used a prospective, single-center, randomized controlled design. A total of 63 pterygium patients (63 affected eyes) and 45 normal healthy controls were enrolled in the Ophthalmology Department, the first affiliated hospital of Anhui Medical University and Changsha Aier Eye Hospital, from September 2017 to November 2018. The study group included 39 women and 24 men, with a mean age of 52.43 ± 6.27 years (range: 38–69 years). The control group included 26 women and 19 men, with a mean age of 50.11 ± 7.78 years (range: 36–68 years).

The patients in the study group had idiopathic pterygium of a single eye. Patients were excluded from participation if they had worn contact lenses within the past 3 months, had experienced ocular injury or surgery, suffered from infectious or allergic conjunctivitis, relied on application of artificial tears, or suffered from any systemic diseases with the potential to interfere in the study outcomes [9].

The following criteria were used to evaluate the patients: the ocular surface disease index (OSDI) questionnaire, NIBUTav, tear meniscus height (TMH), and the Schirmer I Test (SIT). These evaluations were conducted as outlined by the MGD Workshop Report (2011), with slight modifications. A slit lamp was used to assess and grade eyelid margin abnormalities. MG dropout and lipid layer grading were assessed using a Keratograph 5M (Oculus, Wetzlar, Germany). Slit-lamp cameras were used for imaging the participants, after which the pterygium size and thickness were measured. The same ophthalmologist performed all the procedures in a darkened room (Table 1).

### 2.1. OSDI Evaluation

Dry eye symptoms were assessed using the OSDI questionnaire. It was designed to assess quality of life because it pertains to vision in people with dry eye disease. A total of 12 questions about symptoms experienced over the previous week were administered to participants, with possible scores ranging from 0 to 48.

### 2.2. NIBUTav Assessment

For the NIBUTav assessment, the patients were seated facing the Keratograph 5M device with their jaw supported on an appropriate support. A Placido disk containing 22 red concentric circles was then projected onto the patient's eye, and the patient was requested to blink twice while staring at the central spot. While the eye remained open, the NIBUTav value was determined, and appropriate details related to tear break size were displayed on the screen.

### 2.3. SIT

After the patients completed the NIBUTav assessment, they were given a 30-min rest period. The SIT paper was then placed in a region representing one-third of the middle-to-lateral conjunctival sac. The patients were requested to shut their eyes for 5 min, after which the paper was removed. No topical anesthesia was administered for this protocol.

### 2.4. Eyelid Margin Assessment

Eyelid margin abnormalities were evaluated using slit lamp-diffused light with the following scoring: 1 = irregular eyelid margin, 2 = vascular engorgement, 3 = obstructed glandular orifices, and 4 = anterior or posterior mucocutaneous junction displacement. If none of these abnormalities were detected, a score of 0 was given.

### 2.5. Lipid Layer Grading

Using the lipid layer grading program of the Keratograph 5M equipment, the thickness of the lipid layer was divided into the three following levels, according to structural clarity and color richness: thin (level 1), normal (level 2), and thick (level 3). A thin lipid layer structure is fuzzy, with a gray color. A normal lipid layer structure is clear, with a rich color. A thick lipid layer structure is very clear, with an extremely rich color.

### 2.6. Noncontact Infrared Meibography

Patients were seated in front of the Keratograph 5M machine, as described above. Then, the Meibo-Scan Program was used to measure the MG dropout, assigning the following scores as appropriate: 0, no absence; 1, <1/3 of glands absent; 2, >1/3 but <2/3 of glands absent; and 3, >2/3 glands absent. Each eye was assigned a score ranging between 0 and 6, and both eyelids were scored.

### 2.7. Pterygium Assessment

**Figure 1:**
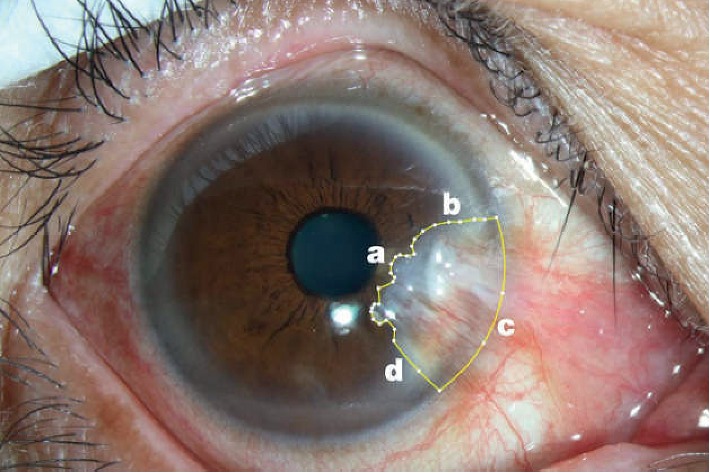
ImageJ software used to calculate the size of the pterygium (a) Edge of the pterygium head; (b) Edge of the upper boundary of the pterygium; (c) Edge of the nasal border of the pterygium, coinciding with the border of the limbus; (d) Edge of the lower boundary of the pterygium.

Anterior segment images were used to assess the pterygium size, after imaging, using a Haag-Streit BQ 900 slit lamp. The ImageJ software program was used to measure the size of the horizontal pterygium length from the limbus to the apex, as well as the size of the corneal pterygium area (Figure 1). The same experienced operator conducted all the measurements [10].

**Figure 2:**
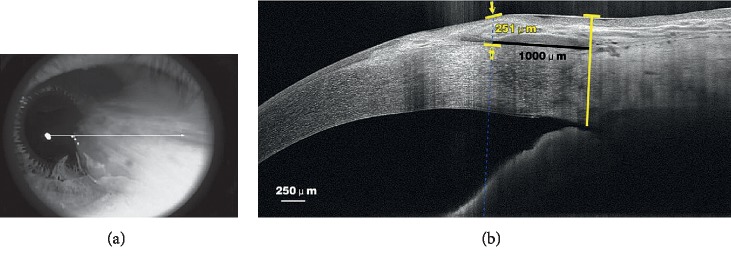
(a) Anterior segment SD-OCT horizontal OCT scan (parallel to the axis of the midpoint of the cornea on the nasal and particular sides) of a primary pterygium. (b) Anterior segment SD-OCT measures the thickness of pterygium at 1 mm in the limbus. The primary pterygium is the overgrown section attached to the cornea. The value of 251 *μ*m represents the thickness of the pterygium at 1 mm in the limbus. The pterygium is present between the two arrows.

The pterygium thickness was measured using anterior segment spectral domain optical coherence tomography (SD-OCT) (RTVue-100, Optovue, Freemont, CA, USA). The CL-line single line scan mode was selected for the anterior segment telephoto lens. The patient's head was adjusted, and the eye was fixed in a still position to the extreme left or right side, with a scan direction of 0–180°. The anterior segment SD-OCT was scanned at the midpoint of the cornea on the nasal and temporal sides of the patient's eye, as shown in Figure 2(a). The front and back of the lens were adjusted to focus the image. Each inspection was continuously scanned three times, with an interval of 3–5 s. Image-Pro Plus 6.0 and Adobe Photoshop CS5 were used to detect the pterygium thickness. To accomplish this, a vertical line was made from the scleral process to the corneal surface, and the thickness of the pterygium at 1 mm from the limbus in the vertical line was measured, as shown in Figure 2(b) [11, 12].

### 2.8. Surgical Technique and Postoperative Care

Subconjunctival anesthesia (20 mg/ml lidocaine HCl, 0.0125 mg/ml epinephrine) was used during surgery. Wescott's scissors were used to cut the pterygium near the limbus; the pterygium head and associated fibrous subjunctival tissue were carefully removed from the cornea using a number 15 blade. Monomial cauterization was used as appropriate. Where suitable, a conjunctival flap was generated using inferomedial conjunctival tissue by preparing the flap from tissue near the limbus and the defect margin. This flap was carefully removed without disrupting Tenon's capsule, and it was then sutured over the site of the defect using 10-0 Vicryl™ sutures.

Bausch + Lomb (Rochester, NY, USA), PureVision (Balafilcon A) Power 0.0 D therapeutic contact lenses (TCLs) were given to all the treated patients, and 0.3% tobramycin and 0.05% dexamethasone eye drops were used to treat the patients' eyes four times daily for 7–10 days following surgery. The sutures and therapeutic contact lenses were removed 1 week after surgery. The patients did not use artificial tears during the study period [13].

### 2.9. Statistical Analysis

SPSS v 20.0 software (SPSS Inc., Chicago, IL, USA) was used for all the analyses. Data are presented as means ± standard deviation (SD), and the groups were compared using *F*-tests, Mann–Whitney *U* tests, and one-way analysis of variance (ANOVA), as appropriate. Pearson's correlation analyses were used to assess the correlations between the variables. The statistically significant threshold was *p* < 0.05.

## 3. Results

The mean pterygium size of the patients in the study group was 34.08 ± 11.12 mm^2^ (range: 16.55–57.78 mm^2^); the mean pterygium thickness was 282.13 ± 92.12 *μ*m (range: 75–434 *μ*m).

### 3.1. Features of Ocular Surface Disorders and MG Abnormalities in the Pterygium Patients

Table 1 presents the relevant parameters pertaining to dry eye and the MG abnormalities in both the pterygium group and the control group. The age and sex ratios were comparable between the groups (*p* > 0.05). Ocular discomfort was the primary complaint among the pterygium patients, with severity levels ranging from mild to severe. The pterygium patients had a significantly elevated OSDI value relative to the controls (20.11 ± 4.27 and 12.00 ± 2.87, respectively; *p* < 0.001). However, the NIBUTav was lower in the pterygium patients than the healthy controls (7.78 ± 3.50 and 10.81 ± 2.77, respectively; *p* < 0.001). The tear volume did not differ significantly between the two groups (11.70 ± 4.36 mm and 12.11 ± 3.27 mm, respectively), nor did the TMH (0.24 ± 0.06 mm and 0.24 ± 0.06 mm, respectively; *p* > 0.05); both groups were in the normal range for these values.

The MG parameters in both groups are also shown in Table 1. The eyelid margin scores, lipid layer grading, and meiboscores were significantly different between the pterygium and control groups (*p* < 0.05). The eyelid margin abnormality scores and meiboscores were markedly elevated in the pterygium group in comparison with the normal controls (*p* < 0.01; Table 1). However, the lipid layer grading was significantly lower in the patients in the pterygium group than the normal controls (*p* < 0.01).

### 3.2. Correlation between the Pterygium Parameters and the Preoperative Ocular Surface Indicators

**Figure 3:**
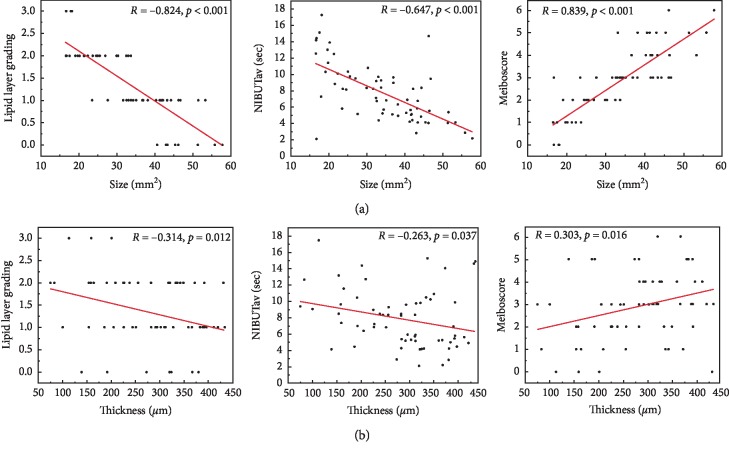
Correlations between pterygium size and thickness and various clinical indicators pre-excision in the pterygium patients: (a) Pterygium size; (b) Pterygium thickness (*R*, Pearson's correlation coefficient, −1 ≤ *R* ≤ 1). A *p* value <0.05 was considered to be statistically significant.

Size and thickness are two of the key parameters used to clinically evaluate pterygium. The size and thickness of the pterygium in the pterygium patients was found to be significantly correlated with the meiboscore (*R* = 0.839, *p* < 0.001; *R* = 0.303, *p*=0.016). These parameters were inversely correlated with NIBUTav (*R* = −0.647, *p* < 0.001; *R* = −0.263, *p*=0.037) and the lipid layer grading (*R* = −0.824, *p* < 0.001; *R* = −0.314, *p*=0.012; Table 2; Figure 3).

### 3.3. Postoperative Ocular Surface Characteristics in the Pterygium Patients

The postoperative dry eye and MG abnormality results for both groups are shown in Table 3. No significant differences were found between the preoperative SIT, TMH, or meiboscore results and the 1-, 3-, and 6-month preoperative results (*p* > 0.05).

As shown in Table 3, the OSDI values, NIBUTav results, and lipid layer grading 1, 3, and 6 months after surgery were significantly different from the preoperative values (*p* < 0.05). Furthermore, the OSDI values, NIBUTav results, and lipid layer grading 3 and 6 months after surgery were significantly different from those 1 month after surgery (*p* < 0.05). Interestingly, no differences were found for the OSDI values, NIBUTav results, and lipid layer grading 3 and 6 months after surgery (*p* < 0.05).

The postoperative eyelid margin abnormality scores were higher than the preoperative scores in the pterygium patients. However, there was no significant difference in the eyelid margin scores obtained 1, 3, and 6 months after surgery (*p* > 0.05).

### 3.4. Correlation between the Pterygium Parameters and Postoperative Ocular Surface Indicators

Pterygium size was significantly negatively correlated with ocular surface indicators 1 month after surgery, including the SIT, TMH, and lipid layer grading values (*R* = −0.950, *p* < 0.001; *R* = −0.934, *p* < 0.001; and *R* = −0.845, *p* < 0.001, respectively). Moreover, these ocular surface indicator parameters were significantly correlated with one another as well as with pterygium thickness (*R* = −0.354, *p*=0.004; *R* = −0.288, *p*=0.022; and *R* = −0.253, *p*=0.045, respectively). In contrast, 3 and 6 months after surgery, no significant differences in these ocular surface indicators were found for either the pterygium size or thickness.

The OSDI values obtained 1, 3, and 6 months after surgery were correlated with pterygium size (*R* = 0.976, *p* < 0.001; *R* = 0.985, *p* < 0.001; and *R* = 0.978, *p* < 0.001, respectively) and thickness (*R* = 0.277, *p*=0.028; *R* = 0.284, *p* = 0.024; and *R* = 0.286, *p* = 0.023, respectively). The NIBUTav values obtained 1, 3, and 6 months after surgery were negatively correlated with pterygium size (*R* = −0.342, *p*=0.007; *R* = −0.430, *p*=0.001; and *R* = −0.342, *p*=0.007, respectively) and thickness (*R* = −0.598, *p* < 0.001; *R* = −0.568, *p* < 0.001; and *R* = −0.598, *p* < 0.001, respectively).

**Figure 4:**
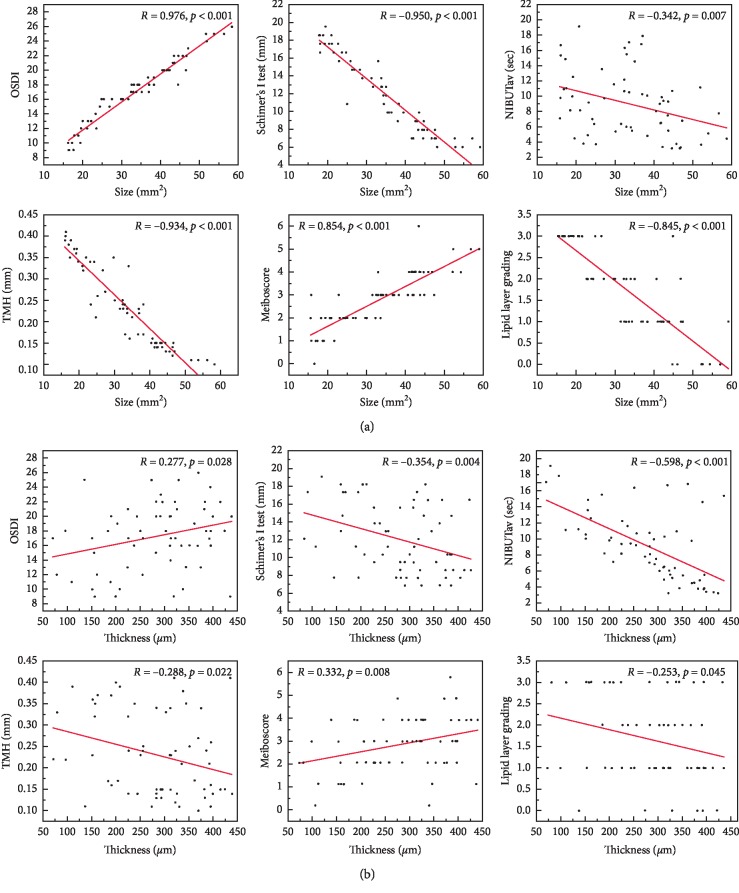
Correlations between pterygium size and thickness and various clinical indicators 1 month after surgery in patients with pterygium: (a) Pterygium size; (b) Pterygium thickness (*R*, Pearson's correlation coefficient, −1 ≤ *R* ≤ 1). A *p* value <0.05 was considered statistically significant.

The pterygium patients' meiboscores were not significantly changed 1, 3, and 6 months after surgery. Accordingly, the meiboscores 1, 3, and 6 months after surgery were significantly correlated with pterygium size (*R* = 0.854, *p* < 0.001; *R* = 0.702, *p* < 0.001; and *R* = 0.882, *p* < 0.001, respectively) and pterygium thickness (*R* = 0.332, *p*=0.008; *R* = 0.284, *p*=0.024; and *R* = 0.299, *p*=0.017, respectively; see Figure 4). No significant correlations were found between the eyelid margin abnormality score and either of the two pterygium parameters.

## 4. Discussion

Postoperative discomfort is a significant concern in patients being treated via pterygium excision, leading many people to decline or postpone surgery. Thus, it is vital to improve a patient's prognosis after surgery [14]. Recently, efforts have been made to detail the relationship between pterygium surgery and tear film function. However, currently, there is no reliable clinical indicator associated with patient prognosis after this type of operation. Moreover, how MGD affects tear film instability in those undergoing pterygium excision or other ocular surgeries remains uncertain [7, 15]. In the present study, postoperatively, the pterygium patients had more severe dry eyes and MGD than the controls, and most of the dry eye and MGD parameters were significantly correlated with the pterygium parameters before and after surgery in these patients. The present study's data suggest that pterygium size and thickness are correlated with ocular surface damage, thereby representing a potential means of evaluating patient prognosis.

This study found that the mean OSDI score was not only statistically significantly reduced in the pterygium group (*p* < 0.05 for the three groups), and it was also correlated with the size and thickness of the pterygium 1 to 6 months after surgery. However, the difference was still significant in comparison with the normal control group at these timepoints. A previous study suggested that tear fluid secretion may increase in order to compensate for MG loss as a means of achieving ocular surface homeostasis [16]. The present study found the tear film quantity in the patients with pterygium to be adequate, but its quality or composition was abnormal. Therefore, it was speculated that changes in MG morphology may be associated with uncomfortable ocular symptoms in patients. Importantly, because a larger pterygium size is associated with greater postoperative discomfort, it is vital that patients be treated as early as possible.

A significant difference in SIT or TMH was not detected between the control participants and the pre- and postoperative pterygium patients, indicating that the tear meniscus production did not change in the pterygium group. The correlation between pterygium and SIT and TMH has been difficult to define. However, a Pearson's correlation analysis showed the pterygium size to be correlated with the 1-month postoperative SIT and TMH values. Conversely, Kampitak and Leelawongtawun [17] demonstrated that the SIT results did not change in pterygium patients, and there was no correlation between pterygium size and the SIT results or the tear breakup time. In the present study, it was speculated that the conjunctiva of patients may be resected too extensively during surgery, leading to damage of the lacrimal caruncle, plica semilunaris, and fornix conjunctiva. This could result in a decrease in goblet cell density and destruction of the lacrimal gland, thereby leading to a decrease in tear film mucin secretion and basal tears that affects the stability of the tear film surface.

The present study found that the NIBUTav value was significantly reduced in the pterygium group, which was consistent with the results previously reported in the literature. A shorter NIBUTav is associated with tear film instability. This study found that the NIBUTav was prolonged 1 month after surgery, which confirmed that, to a certain extent, pterygium excision surgery can restore a patient's tear function. 3 and 6 months after surgery, NIBUTav in the pterygium group returned to the normal group levels, and the tear membrane breakup time improved significantly. Moreover, this study's data revealed that the preoperative NIBUTav values were inversely correlated with the pterygium thickness, while the NIBUTav values 1 month after surgery were inversely related to the size index. It was speculated that the pterygium affected the regularity and smoothness of the eyeball surface, thereby affecting the normal distribution of tears, leading to instability of the tear film. Inflammation of the eyeball surface is relieved after surgical removal of the tendon tissue. With the gradual repair of corneal epithelial damage, the tear film function can gradually return to normal.

The tear film consists of three layers. The most superficial layer is the lipid layer, which is produced by the MGs; this layer stabilizes the tear film by retarding evaporation and lowering surface tension [18, 19]. The present study's results suggest that the lipid layer grading was significantly reduced in the pterygium group. The pterygium size and thickness were significantly negatively correlated with the preoperative lipid layer grading. The reduction in lipid layer grading in patients with pterygium may be related to two factors. On the one hand, an irregular ocular surface structure may result in an uneven distribution and decreased adhesion of the lipid layers; on the other hand, corneal sensation loss and blink reduction may lead to decreased secretion of lipids in the MGs. At 1, 3, and 6 months postoperatively, the thickness of the lipid layer grading increased significantly after surgery in comparison with the preoperative levels (1.32 ± 0.76), for thicknesses of 1.67 ± 0.99, 2.00 ± 0.65, and 2.05 ± 0.71, respectively. These differences were significant (Table 3). The lipid layer grading was restored to the normal level 3 months after surgery (*p* > 0.05). At that time, the pterygium patients' NIBUTav values were also significantly improved, indicating that improvement in the lipid layer played an important role in maintaining the tear film stability of the ocular surface. Therefore, it was speculated that the quantity of the tear film in the patients with pterygium was adequate, but its quality or composition was abnormal.

The associations between MG morphology and the pterygium parameters were also investigated using a noncontact meibographic technique. The resulting data demonstrated that the MG loss was more significant in the pterygium patients than the healthy controls. A correlation analysis confirmed that both pterygium size and thickness were positively correlated with the meiboscore. In fact, there is the potential for direct contact between the hypertrophic pterygium and palpebral conjunctiva, leading to compression beneath the MGs over an extended period of time. This suggests that pterygium can drive different degrees of MG loss as the disease progresses. After pterygium surgery, no changes were observed in the morphology of the MGs. The atrophy, loss, and bending of the MGs were difficult to relieve via surgery. The differences in the meiboscore values between the preoperative and postoperative timepoints were not statistically significant (Table 3). However, this does not mean that growth may have occurred if the study had been conducted beyond 6 months because compensatory growth of the ducts and acinus may take a long time.

The eyelid margin abnormality score was found to be significantly increased in the pterygium group. After surgery, the eyelid margin score was reduced, but it was still higher in the pterygium group than the normal controls. Previous studies have revealed that pterygia is characterized by an inflammatory infiltrate with a prominent vascular reaction [20]. Chronic repeated inflammation may cause meibum stagnation and MG keratinization. After pterygium excision, this limbal microenvironmental anomaly was improved. Nevertheless, the hyperkeratinization of the epithelium at the eyelid margin and MG may cause structural changes within the MGs [21, 22].

Most previous studies have focused on the relationship between pterygium and tear film dynamics [23]. However, previous studies did not assess how the pterygium parameters are associated with patient prognosis after surgery. The present study compared tear function changes before and after pterygium excision, and the functions were found to be partially restored after surgery; OSDI, NIBUTav, eyelid margin abnormality, and lipid layer grading all improved (Table 4). Thus, after development, pterygium can directly drive abnormal tear film function and MGD. This study also found that the pterygium size and thickness values were significantly correlated with most of the parameters (such as ocular surface comfort, tear film stability, and MG function) in the pterygium patients before and after surgery. A large and thick pterygium may have aggravated the tear stability and ocular surface damage, potentially leading to a shorter tear film breakup time, thin lipid layers, and extensive MG loss. Furthermore, the pterygium size was negatively correlated with the SIT, TMH, NIBUTav, and lipid layer grading results at different timepoints after surgery. Nevertheless, no association was found between the pterygium parameters and the long-term outcomes of excision surgery. Thus, it is speculated that a large pterygium size may be a risk factor for dry eye formation and MGD 1 month after pterygia surgery. To minimize MG loss and postoperative discomfort, surgical treatment should be conducted as early as possible.

## 5. Conclusion

In conclusion, to the best of our knowledge, this study is the first to focus on unraveling the correlation between pterygium parameters and ocular surface comfort, tear film stability, and MG function before and after surgery. Thus, the study provides a novel strategy for clinical assessment of the prognosis of patients following pterygium excision surgery.

## Figures and Tables

**Table 1:** Features of ocular surface disorders and MGs in the pterygium patients.

	Controls (*n* = 45)	Pterygium group (*n* = 63)	*p*
Age	50.11 ± 7.78	52.43 ± 6.27	0.090
Sex ratio (F/M)	26/19	39/24	0.666
OSDI	12.00 ± 2.87	20.11 ± 4.27	<0.001
SIT (mm)	12.11 ± 3.27	11.70 ± 4.36	0.575
NIBUTav (s)	10.81 ± 2.77	7.78 ± 3.50	<0.001
TMH (mm)	0.24 ± 0.06	0.24 ± 0.06	0.688
Meiboscore	1.02 ± 0.69	2.91 ± 1.51	<0.001
Eyelid margin abnormality	1.04 ± 0.74	1.48 ± 0.84	0.007
Lipid layer grading	2.11 ± 0.75	1.32 ± 0.76	<0.001

*p*, significance level in the Pearson's correlation analysis. Data are expressed as means ± standard deviation (SD).

**Table 2:** Correlations between the pterygium parameters, dry eye indices, and meibomian gland functionality.

	Size		Thickness	
	*R*	*p*	*R*	*p*
OSDI	0.216	0.089	−0.022	0.862
SIT (mm)	0.073	0.570	0.045	0.727
NIBUTav (s)	−0.647	<0.001	−0.263	0.037
TMH (mm)	−0.109	0.395	0.122	0.342
Meiboscore	0.839	<0.001	0.303	0.016
Eyelid margin abnormality	0.197	0.123	0.007	0.960
Lipid layer grading	−0.824	<0.001	−0.314	0.012

*R*, Pearson's correlation analysis correlation value. *p*, Pearson's correlation analysis significance value.

**Table 3:** Ocular surface disorders and MGs in the pterygium patients after excision surgery.

	1 month after surgery	3 months after surgery	6 months after surgery	*p*	*p*		
					2 and 1	3 and 1	3 and 2
OSDI	17.25 ± 4.48^*∗*^	14.51 ± 4.01^*∗*^	14.40 ± 4.15^*∗*^	<0.001	<0.001	<0.001	0.818
SIT (mm)	11.92 ± 4.31^#†^	12.68 ± 3.68^#†^	12.91 ± 3.31^#†^	0.241	0.273	0.158	0.749
NIBUTav (s)	9.04 ± 4.06^*∗*^	11.12 ± 4.12^*∗*^^†^	11.14 ± 4.27^*∗*^^†^	<0.001	<0.001	<0.001	0.972
TMH (mm)	0.23 ± 0.10^#†^	0.23 ± 0.09^#†^	0.23 ± 0.09^#†^	0.882	0.846	0.967	0.815
Meiboscore	2.86 ± 1.19^#^	2.94 ± 1.05^#^	2.79 ± 1.25^#^	0.582	0.466	0.560	0.191
Eyelid margin abnormality	1.10 ± 0.61^*∗*^^†^	1.10 ± 0.59^*∗*^^†^	1.10 ± 0.59^*∗*^^†^	<0.001	1.000	1.000	1.000
Lipid layer grading	1.67 ± 0.99^*∗*^	2.00 ± 0.65^*∗*^^†^	2.05 ± 0.71^*∗*^^†^	<0.001	0.005	0.001	0.684

*p*, significance level in Pearson's correlation analysis. Data are expressed as means ± SD. ^**#**^Preoperative vs. postoperative comparison; *p* > 0.05. ^*∗*^Preoperative vs. postoperative comparison; *p* < 0.05. ^†^Preoperative vs. control group comparison; *p* > 0.05.

**Table 4:** Correlations between the pterygium parameters, dry eye indices, and MG functionality postoperatively.

	1 month after surgery				3 months after surgery				6 months after surgery			
	Size		Thickness		Size		Thickness		Size		Thickness	
	*R*	*p*	*R*	*p*	*R*	*p*	*R*	*p*	*R*	*p*	*R*	*p*
OSDI	0.976	<0.001	0.277	0.028	0.985	<0.001	0.284	0.024	0.978	<0.001	0.286	0.023
SIT (mm)	−0.950	<0.001	−0.354	0.004	−0.100	0.436	−0.167	0.190	0.004	0.974	−0.230	0.070
NIBUTav (s)	−0.342	0.007	−0.598	<0.001	−0.430	0.001	−0.568	<0.001	−0.342	0.007	−0.598	<0.001
TMH (mm)	−0.934	<0.001	−0.288	0.022	0.131	0.308	0.056	0.665	0.009	0.945	0.135	0.293
Meiboscore	0.854	<0.001	0.332	0.008	0.702	<0.001	0.284	0.024	0.882	<0.001	0.299	0.017
Eyelid margin abnormality	0.183	0.151	−0.023	0.856	0.208	0.101	0.008	0.953	0.223	0.079	−0.054	0.627
Lipid layer grading	−0.845	<0.001	−0.253	0.045	−0.105	0.412	−0.046	0.721	−0.150	0.240	−0.100	0.437

*R*, Pearson's correlation analysis coefficient value; *p*, Pearson's correlation analysis significance value.

## Data Availability

Our article is about clinical research on ocular surface diseases. All data are obtained through our clinical observation and testing. The clinical data used to support the findings of this study are included within the article. The raw data used to support the findings of this study are available from the corresponding author upon request.
